# 0853. Elevated levels of soluble rage predict impaired alveolar fluid clearance in a translational mouse model of acute respiratory distress syndrome (ARDS)

**DOI:** 10.1186/2197-425X-2-S1-P62

**Published:** 2014-09-26

**Authors:** R Blondonnet, M Jabaudon, G Clairefond, J Audard, D Bouvier, G Marceau, P Blanc, P Dechelotte, V Sapin, J-M Constantin

**Affiliations:** R2D2 - EA 7281, School of Medicine, Universite d'Auvergne Clermont-Ferrand I, Clermont-Ferrand, France; Intensive Care Unit, Department of Anesthesiology and Critical Care Medicine, Estaing University Hospital, CHU Clermont-Ferrand, Clermont-Ferrand, France; Department of Medical Biochemistry and Molecular Biology, Estaing University Hospital CHU Clermont-Ferrand, Clermont-Ferrand, France; Department of Pathology, Estaing University Hospital CHU Clermont-Ferrand, Clermont-Ferrand, France

## Introduction

Receptor for advanced glycation endproducts (RAGE) is a transmembrane pattern-recognition receptor of the immunoglobulin superfamily that is abundantly expressed in the lung and primarily located on the basal surface of alveolar type I cells. RAGE is implicated in ARDS as an important pathway to alveolar inflammation and, when its soluble form sRAGE is assayed in plasma or pulmonary edema fluid, as a marker of AT I cell injury [1]. Functional activity of AT 1 cells can be assessed by the measurement of alveolar fluid clearance (AFC) rate [2], but the relationship between sRAGE plasma levels of sRAGE and AFC rates has never been investigated.

## Objectives

To report plasma levels of sRAGE in a translational mouse model of direct acid-induced epithelial injury, and to test their correlation with AFC rates.

## Methods

Forty-one male CD-1 mice were divided in 2 groups: an “HCl” group of mice who received a tracheal instillation of hydrochloric acid on day 0, and a group of control uninjured animals. Mice were evaluated on day 0, day 1, day 2 and day 4 after a 30-minute period of mechanical ventilation : after sacrifice, blood and undiluted lung edema fluid (EF) were sampled. Before initiation of MV, all mice received a tracheal instillation of bovine serum albumin (BSA 5%) in order to detect changes in alveolar protein levels over 30 minutes. Plasma levels of sRAGE and total protein levels were measured in duplicate by ELISA and colorimetric detection, respectively. AFC rate values were corrected after measurement of mouse serum albumin in EF.

## Results

Basal AFC rate was 35% over 30 min in HCl-injured mice, but it was significantly depressed on day 1 (16% over 30 min; p=0.02). Over time, AFC reached basal levels again. Plasma levels of sRAGE were higher in HCl-treated animals than in control animals on day 1 (p=0.03) and day 2 (p=0.02). Significant correlation was found between AFC rates and plasma levels of sRAGE (Spearman correlation coefficient -0.49 (IC 95 [-0.70 ; -0.19] p=0.04)).

**Figure 1:**
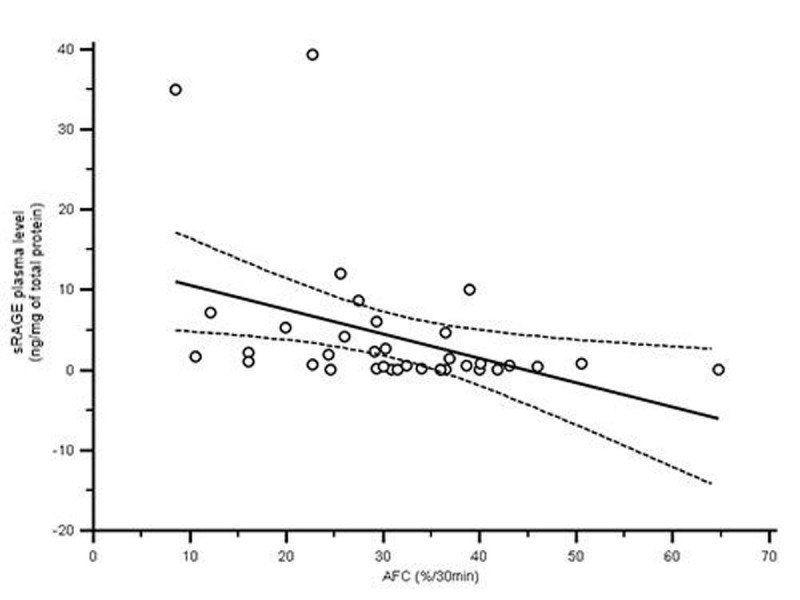
Relation between AFC rates and sRAGE plasma levels

## Conclusions

The highest impairment in AFC is reported on day 1 in our animal model of acid-induced injury. sRAGE levels are also higher in injured mice and may be a good surrogate marker of AT I cell injury. This newly decribed relationship between AFC rates and sRAGE plasma level in a mouse model of direct epithelial injury confirms previous results from an ex vivo model of isolated human uninjured lungs [3]. Our results support further translational investigation on the role of RAGE in alveolar injury and recovery.
